# Supplementary material for: Single-Cell mRNA Analysis for the Identification of Molecular Pathways of IRF1 in HER2+ Breast Cancer
Source: Cells. 2025 Aug 13;14(16):1246. doi: 10.3390/cells14161246 (PMC12384442; doi:10.3390/cells14161246)
Supplement: Supplementary file 1 [file cells-14-01246-s001.zip › cells-3618023-supplementary.pdf]

TABLES as WORD Editable Document

Table S1

**pathway**	**pval**	**padj**
HALLMARK_OXIDATIVE_PHOSPHORYLATION	0,001040583	0,00794155
HALLMARK_MYC_TARGET	0,00104612	0,00794155
HALLMARK_ DNA_REPAIR	0,001108647	0,00794155
HALLMARK_ REACTIVE_OXIGEN_SPECIES_PATHWAY	0,001270648	0,00794155
HALLMARK_ADIPOGENESIS	0,001072961	0,00794155
HALLMARK_ FATTY ACID METABOLISM	0,00116195	0,009238729
HALLMARK_UNFOLDED_PROTEIN_RESPONSE	0,001146789	0,00794155
HALLMARK_XENOBIOTIC_METABOLISM	0,002107802	0,009238729
HALLMARK_MTORC1_SIGNALING	0.002162162	0,009238729
HALLMARK_E2F_TARGETS	0,002202643	0,009238729
HALLMARK_UV RESPONSE_UP	0,002217295	0,009238729
HALLMARK_COAGULATION	0,001183432	0,00794155
HALLMARK_GLICOLYSIS	0,004338395	0,015494267
HALLMARK_HYPOXIA	0,00867679	0,016287
HALLMARK_MYOGENESIS	0,01818188	0,037037037
HALLMARK_MYC_TARGETS_V2	0,016290727	0,037024379
HALLMARK_P53_PATHWAY	0,023809524	0,044091711
HALLMARK_PEROXISOME	0,025	0,044642857

Table S2

**pathway**	**pval**	**padj**
HALLMARK_OXIDATIVE_PHOSPHORYLATION	0,00103	0,007135
HALLMARK_EPITHELIAL_MESENCHYMAL_TRANSITION	0,001031	0,007135
HALLMARK_INTERFERON_ALPHA_RESPONSE	0,001142	0,007135
HALLMARK_DNA_REPAIR	0,001063	0,007135
HALLMARK_COAGULATION	0,01142	0,007135
HALLMARK_GLYCOLYSIS	0,001062	0,007135
HALLMARK_ INTERFERON_GAMMA_RESPONSE	0,001044	0,007135
HALLMARK_APICAL JUNCTION	0,001089	0,007135
HALLMARK_ADIPOGENESIS	0,002079	0,010893
HALLMARK_FATTY ACID METABOLISM	0,002179	0,010893
HALLMARK_ANGIOGENESIS	0,011952	0,037707

HALLMARK_P53_PATHWAY	0,003128	0,013034
HALLMARK_MYC_TARGETS_V1	0,003093	0,013034
HALLMARK_HYPOXIA	0,019958	0,049895
HALLMARK_ALLOGRAFT_REJECTION	0,012586	0,037707

Table S3 (left side)

**RA CXCL12 Synovial Fibroblast Pathway = FIGURE2**	**pval**	**padj**
**HALLMARK_OXIDATIVE_PHOSPHORYLATION**	**0,001040583**	**0,00794155**
HALLMARK_MYC_TARGET	0,00104612	0,00794155
**HALLMARK_ DNA_REPAIR**	0,001108647	0,00794155
HALLMARK_ REACTIVE_OXIGEN_SPECIES_PATHWAY	0,001270648	0,00794155
**HALLMARK_ADIPOGENESIS**	**0,001072961**	**0,00794155**
**HALLMARK_ FATTY ACID METABOLISM**	**0,00116195**	**0,009238729**
HALLMARK_UNFOLDED_PROTEIN_RESPONSE	0,001146789	0,00794155
HALLMARK_XENOBIOTIC_METABOLISM	0,002107802	0,009238729
HALLMARK_MTORC1_SIGNALING	0.002162162	0,009238729
HALLMARK_E2F_TARGETS	0,002202643	0,009238729
HALLMARK_UV RESPONSE_UP	0,002217295	0,009238729
**HALLMARK_COAGULATION**	**0,001183432**	**0,00794155**
**HALLMARK_GLICOLYSIS**	**0,004338395**	**0,015494267**
**HALLMARK_HYPOXIA**	**0,00867679**	**0,016287**
HALLMARK_MYOGENESIS	0,01818188	0,037037037
**HALLMARK_MYC_TARGETS_V2**	**0,016290727**	**0,037024379**
**HALLMARK_P53_PATHWAY**	**0,023809524**	**0,044091711**
HALLMARK_PEROXISOME	0,025	0,044642857

Table S3 (right side)

**HER2+ Fb2 Fibroblast Pathway**	**pval**	**padj**
**HALLMARK_OXIDATIVE_PHOSPHORYLATION**	**0,00103**	**0,007135**
HALLMARK_EPITHELIAL_MESENCHYMAL_TRANSITION	0,001031	0,007135
HALLMARK_INTERFERON_ALPHA_RESPONSE	0,001142	0,007135
**HALLMARK_DNA_REPAIR**	0,001063	0,007135
**HALLMARK_COAGULATION**	**0,01142**	**0,007135**
**HALLMARK_GLYCOLYSIS**	**0,001062**	**0,007135**
HALLMARK_ INTERFERON_GAMMA_RESPONSE	0,001044	0,007135
HALLMARK_APICAL JUNCTION	0,001089	0,007135

**HALLMARK_ADIPOGENESIS**	**0,002079**	**0,010893**
**HALLMARK_FATTY ACID METABOLISM**	**0,002179**	**0,010893**
HALLMARK_ANGIOGENESIS	0,011952	0,037707
**HALLMARK_P53_PATHWAY**	**0,003128**	**0,013034**
**HALLMARK_MYC_TARGETS_V1**	**0,003093**	**0,013034**
**HALLMARK_HYPOXIA**	**0,019958**	**0,049895**
HALLMARK_ALLOGRAFT_REJECTION	0,012586	0,037707

Table S4

Basal cell pathway	pval	padj
HALLMARK_OXIDATIVE_PHOSPHORYLATION	0,001059	0,006127
HALLMARK_INTERFERON_ALPHA_RESPONSE	0,001131	0,006127
HALLMARK_INTERFERON_GAMMA_RESPONSE	0,001053	0,006127
HALLMARK_TNFA_SIGNALING_VIA_NFKB	0,001032	0,006127
HALLMARK_APOPTOSIS	0,001089	0,006127
HALLMARK_COAGULATION	0,001144	0,006127
HALLMARK_P53_PATHWAY	0,001042	0,006127
HALLMARK_ALLOGRAFT_REJECTION	0,001115	0,006127
HALLMARK_ADIPOGENESIS	0,001059	0,006127
HALLMARK_UV RESPONSE_UP	0,00216	0,008999
HALLMARK_COMPLEMENT	0,002146	0,008999
HALLMARK_FATTY_ ACID_ METABOLISM	0,003341	0,011136
HALLMARK_ DNA_REPAIR	0,003236	0,011136
HALLMARK_ REACTIVE_OXIGEN_SPECIES_PATHWAY	0,001225	0,006127
HALLMARK_INFLAMMATORY_RESPONSE	0,003304	0,011136
HALLMARK_IL6_JAK_STAT3_SIGNALING	0,003695	0,011546
HALLMARK_XENOBIOTIC_METABOLISM	0,013187	0,0333333
HALLMARK_CHOLESTEROL_HOMEOSTASIS	0,008343	0,024539
HALLMARK_HYPOXIA	0,016949	0,037481

Table S5 (left side)

Basal cell pathway	pval	padj
**HALLMARK_OXIDATIVE_PHOSPHORYLATION**	**0,001059**	**0,006127**
**HALLMARK_INTERFERON_ALPHA_RESPONSE**	**0,001131**	**0,006127**
**HALLMARK_INTERFERON_GAMMA_RESPONSE**	**0,001053**	**0,006127**

HALLMARK_TNFA_SIGNALING_VIA_NFKB	0,001032	0,006127
HALLMARK_APOPTOSIS	0,001089	0,006127
**HALLMARK_COAGULATION**	**0,001144**	**0,006127**
**HALLMARK_P53_PATHWAY**	**0,001042**	**0,006127**
HALLMARK_ALLOGRAFT_REJECTION	0,001115	0,006127
**HALLMARK_ADIPOGENESIS**	**0,001059**	**0,006127**
HALLMARK_UV RESPONSE_UP	0,00216	0,008999
HALLMARK_COMPLEMENT	0,002146	0,008999
**HALLMARK_FATTY_ ACID_ METABOLISM**	**0,003341**	**0,011136**
HALLMARK_ DNA_REPAIR	0,003236	0,011136
HALLMARK_ REACTIVE_OXIGEN_SPECIES_PATHWAY	0,001225	0,006127
HALLMARK_INFLAMMATORY_RESPONSE	0,003304	0,011136
HALLMARK_IL6_JAK_STAT3_SIGNALING	0,003695	0,011546
HALLMARK_XENOBIOTIC_METABOLISM	0,013187	0,0333333
HALLMARK_CHOLESTEROL_HOMEOSTASIS	0,008343	0,024539
**HALLMARK_HYPOXIA**	**0,016949**	**0,037481**

Table S5 (right side)

**Basal cell cluster pathway**	**pval**	**padj**
**HALLMARK_OXIDATIVE_PHOSPHORYLATION**	**0,00103**	**0,007135**
HALLMARK_EPITHELIAL_MESENKIMAL_TRANSITION	0,001031	0,007135
**HALLMARK_INTERFERON_ALPHA_RESPONSE**	**0,001142**	**0,007135**
HALLMARK_ DNA_REPAIR	0,001063	0,007135
**HALLMARK_ COAGULATION**	**0,001142**	**0,007135**
HALLMARK_GLYCOLYSIS	0,001062	0,007135
**HALLMARK_INTERFERON_GAMMA_RESPONSE**	**0,001044**	**0,007135**
HALLMARK_APICAL_JUNCTION	0,001089	0,007135
**HALLMARK_ADIPOGENESIS**	**0,002079**	**0,010893**
**HALLMARK_ FATTY_ ACID_ METABOLISM**	**0,002179**	**0,010893**
HALLMARK_ANGIOGENESIS	0,011952	0,037707
**HALLMARK_P53_PATHWAY**	**0,003128**	**0,013034**
HALLMARK_MYC_TARGETS	0,003093	0,013034
**HALLMARK_HYPOXIA**	**0,019958**	**0,049896**
HALLMARK_ ALLOGRAFT_REJECTION	0,012588	0,037707

Table S6 (left side)

**HER2+ Fb2 Fibroblast Pathway**	**pval**	**padj**
**HALLMARK_OXIDATIVE_PHOSPHORYLATION**	**0,00103**	**0,007135**
**HALLMARK_EPITHELIAL_MESENCHYMAL_TRANSITION**	**0,001031**	**0,007135**
**HALLMARK_INTERFERON_ALPHA_RESPONSE**	**0,001142**	**0,007135**
**HALLMARK_DNA_REPAIR**	**0,001063**	**0,007135**
HALLMARK_COAGULATION	0,01142	0,007135
**HALLMARK_GLYCOLYSIS**	**0,001062**	**0,007135**
**HALLMARK_ INTERFERON_GAMMA_RESPONSE**	**0,001044**	**0,007135**
HALLMARK_APICAL JUNCTION	0,001089	0,007135
HALLMARK_ADIPOGENESIS	0,002079	0,010893
HALLMARK_FATTY ACID METABOLISM	0,002179	0,010893
HALLMARK_ANGIOGENESIS	0,011952	0,037707
**HALLMARK_P53_PATHWAY**	**0,003128**	**0,013034**
**HALLMARK_MYC_TARGETS_V1**	**0,003093**	**0,013034**
**HALLMARK_HYPOXIA**	**0,019958**	**0,049895**
**HALLMARK_ALLOGRAFT_REJECTION**	**0,012586**	**0,037707**

Table S6 (right side)

**UNC1 Cell Cluster Pathways**	**pval**	**padj**
**HALLMARK_OXIDATIVE_PHOSPHORYLATION**	**0,001148**	**0,012903**
**HALLMARK_INTERFERON GAMMA RESPONSE**	**0,001183**	**0,012903**
**HALLMARK_HYPOXIA**	0,001229	0,012903
**HALLMARK_ALLOGRAFT_REJECTION**	0,01255	0,012903
**HALLMARK_INTERFERON_ALPHA_RESPONSE**	**0,00129**	**0,012903**
**HALLMARK_MYC_TARGETS**	0,002222	0,015152
**HALLMARK_P53_PATHWAY**	0,002424	0,015152
HALLMARK_TNFA_SIGNALING_VIA_NFKB	0,002317	0,015152
HALLMARK_DNA_REPAIR	0,004957	0,027793
HALLMARK_ REACTIVE_OXIGEN_SPECIES_PATHWAY	0,007267	0,027793
**HALLMARK_GLYCOLYSIS**	**0,007782**	**0,027793**
HALLMARK_INFLAMMATORY_RESPONSE	0,013263	0,009384
HALLMARK_KRAS_SIGNALING_UP	0,013459	0,039585
HALLMARK_IL6_JAK_STAT3_SIGNALING	0,012931	0,039585

Table S7 (left side)

**B Cell Cluster Pathways**	**pval**	**padj**
**HALLMARK_ INTERFERON_GAMMA_RESPONSE**	**0,001182**	**0,009411**
**HALLMARK_TNFA_SIGNALING_VIA_NFKB**	0,001135	0,009411
**HALLMARK_ALLOGRAFT_REJECTION**	**0,01202**	**0,009411**
**HALLMARK_INTERFERON_ALPHA_RESPONSE**	0,001318	0,009411
**HALLMARK_UV_RESPONSE_UP**	0,001252	0,009411
**HALLMARK_MTORC1_SIGNALING**	0,001145	0,009411
**HALLMARK_HYPOXIA**	**0,001186**	**0,009411**
**HALLMARK_INFLAMMATORY_RESPONSE**	**0,002635**	**0,016469**
HALLMARK_FATTY_ACID_METABOLISM	0,004087	0,022707
**HALLMARK_APOPTOSIS**	**0,007151**	**0,034722**
**HALLMARK_IL2_STAT5_SIGNALING**	**0,008235**	**0,034722**

Table S7 (left side bottom)

**Plasma Cell Cluster Pathways**	**pval**	**padj**
**HALLMARK_INTERFERON_ALPHA_RESPONSE**	**0,00137**	**0,034247**
**HALLMARK_ALLOGRAFT_REJECTION**	**0,001252**	**0,034247**

Table S7 (right side)

**Natural Killer Cell Cluster Pathways**	**pval**	**padj**
**HALLMARK_ALLOGRAFT_REJECTION**	**0,01189**	**0,005746**
**HALLMARK_INTERFERON GAMMA RESPONSE**	**0,001193**	**0,005746**
**HALLMARK_TNFA_SIGNALING_VIA_NFKB**	**0,001155**	**0,005746**
**HALLMARK_INTERFERON_ALPHA_RESPONSE**	**0,001264**	**0,005746**
**HALLMARK_UV_RESPONSE_UP**	0,001233	0,005746
**HALLMARK_IL2_STAT5_SIGNALING**	**0,001192**	**0,005746**
**HALLMARK_MTORC1_SIGNALING**	0,001166	0,005746
**HALLMARK_APOPTOSIS**	**0,001193**	**0,005746**
**HALLMARK_INFLAMMATORY_RESPONSE**	**0,001252**	**0,005746**
HALLMARK_OXIDATIVE_PHOSPHORYLATION	0,001193	0,005746
**HALLMARK_HYPOXIA**	**0,001221**	**0,005746**
HALLMARK_COMPLEMENT	0,004969	0,019111
HALLMARK_P53_PATHWAY	0,005967	0,020704
HALLMARK_IL6_JAK_STAT3_SIGNALING	0,015048	0,03762
HALLMARK_BILE_ACID_METABOLISM	0,01385	0,036448

Supplementary Material

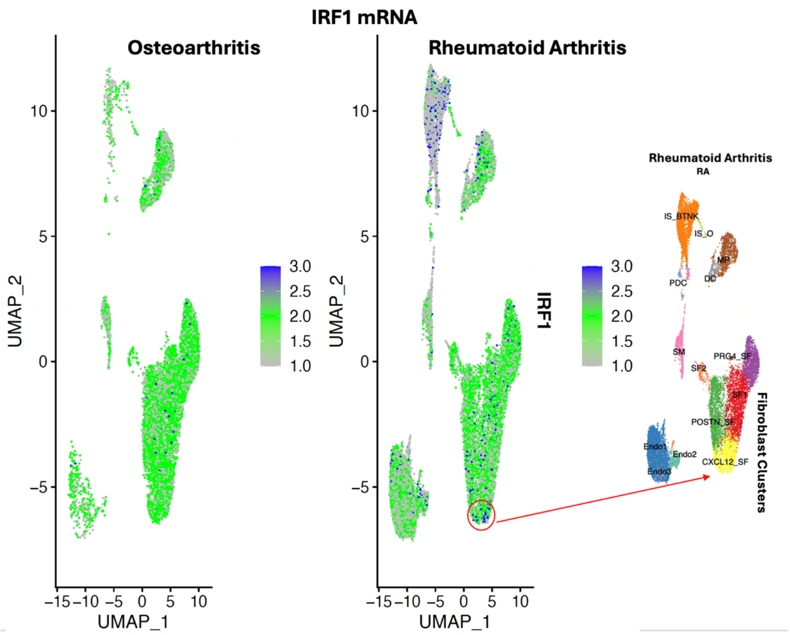

Figure S1.

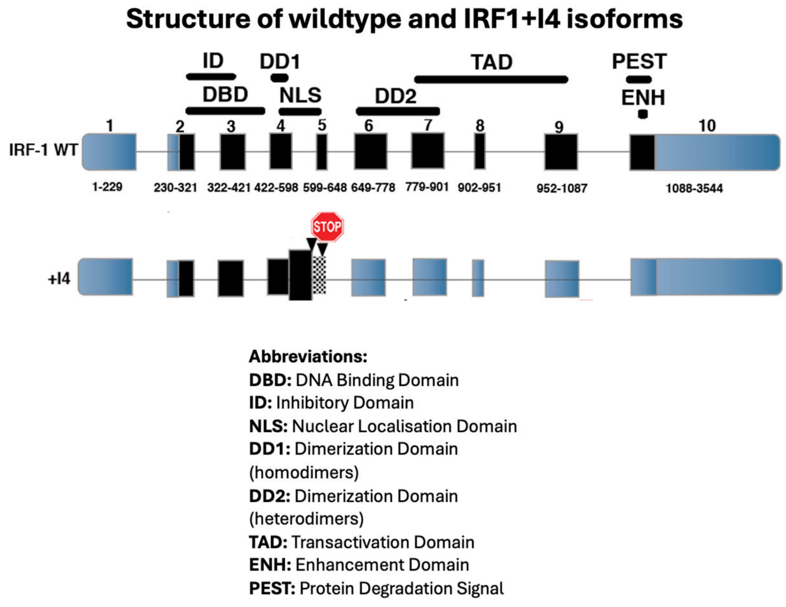

Figure S2.

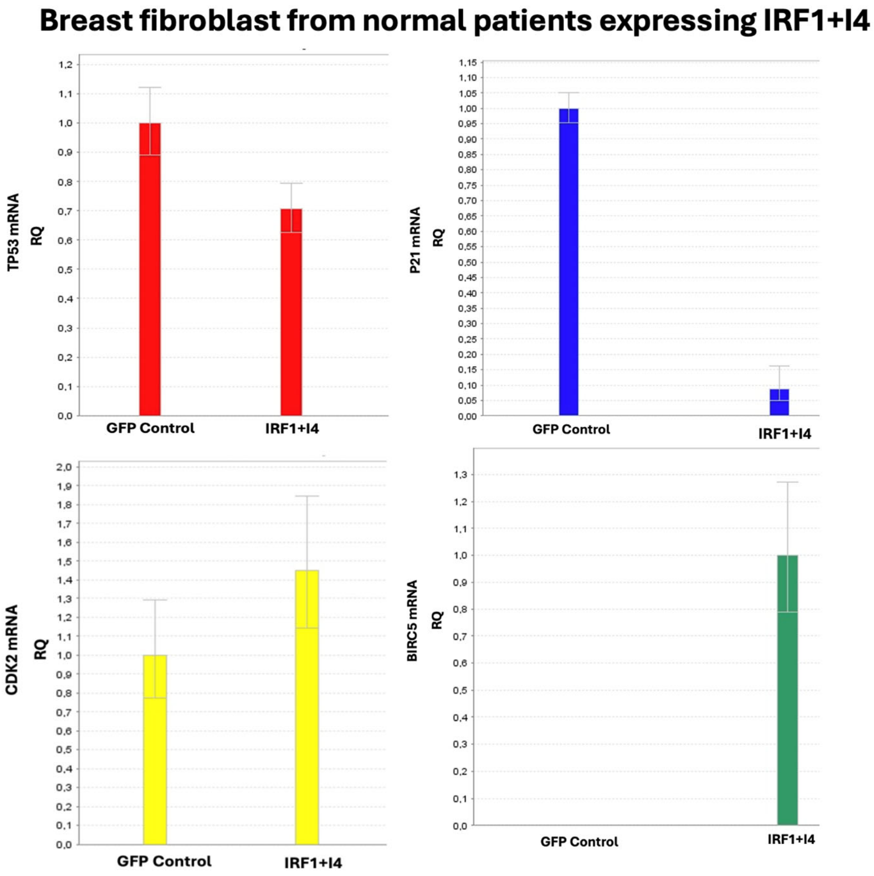

Breast fibroblast from normal patients expressing IRF1+I4
TP53 mRNA RQ
P21 mRNA RQ
CDK2 mRNA RQ
BIRC5 mRNA RQ
GFP Control
IRF1+I4

Figure S3.

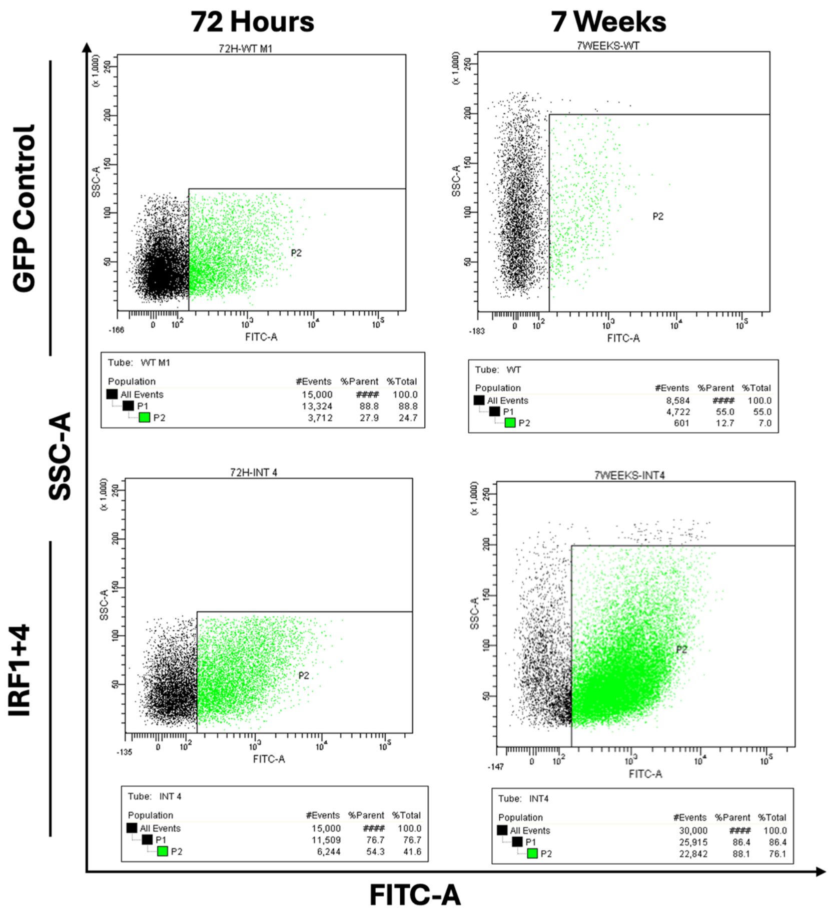

Figure S4.

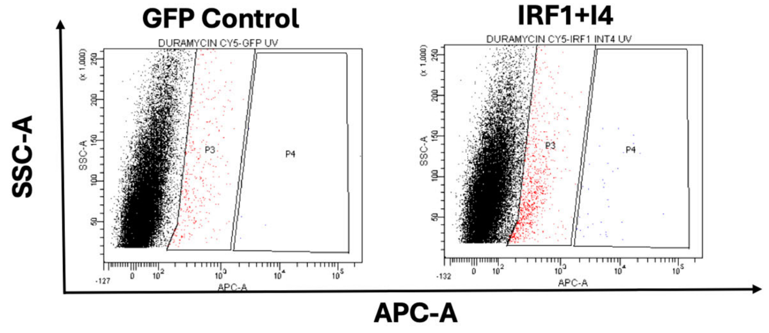

Tube: GFP UV

Population	#Events	%Parent	%Total
All Events	30,000	####	100.0
P1	25,861	86.2	86.2
P3	418	1.6	1.4
P2	24,678	95.4	82.3
P4	4	0.0	0.0
Q1	24,646	95.3	82.2
Q2	645	2.5	2.2
Q3	527	2.0	1.8
Q4	43	0.2	0.1

Tube: IRF1 INT4 UV

Population	#Events	%Parent	%Total
All Events	27,567	####	100.0
P1	24,879	90.2	90.2
P3	945	3.8	3.4
P2	18,280	73.5	66.3
P4	33	0.1	0.1
Q1	18,210	73.2	66.1
Q2	412	1.7	1.5
Q3	5,501	22.1	20.0
Q4	756	3.0	2.7

Flow cytometry assay of breast fibroblast cells apoptosis induced by UV-C treatment. Cells overexpressing IRF-1 isoforms were treated with 50 J/m2 UV-C. Cy5-flurophores and Duramycin were used to measure apoptotic cells during the early stages of apoptosis. IRF-1 WT cells result to be the most affected (P3 Population).

Figure S5.

Supplementary Material - Primers for Quantitative real time PCR

Gene	Gene ID	Primer Fwd Sequence 5'→3'	Primer Rev Sequence 5'→3'
P21 (CDKN1A)	1026	TGGAGACTCTCAGGGTCGAAA	GGCGTTTGGAGTGGTAGAAATC
CDK2	1017	CCTCATCAAGAGCTATCTGTTCCA	ACCCGATGAGAATGGCAGAA
CCNE1	898	TGCGAGCAATTCTTCTGGATT	CAACATTTTCTTGTGTCGCCAT
Tp53	7157	TCAACAAGATGTTTTGCCAACTG	ATGTGCTGTGACTGCTTGTAGATG
BIRC5	332	GGACCACCGCATCTCTACAT	AGTCTGGCTCGTTCTCAGTG
HPRT1	3251	TTTGCTGACCTGCTGGATTACA	GGTCATTACAATAGCTCTTCAGTCTGAT

Supplementary Material. Patient cohort information

normal breast tissue samples (GSE235326)

(https://www.ncbi.nlm.nih.gov/geo/query/acc.cgi?acc= GSE19566 data subset GSE235326)

	Age	menopause status	Tissue source	parity	Etnicity	Treatment
GSM7500385hbcac76	> 50	pre	Reduction Mammoplasty	0	caucasian	none
GSM7500386hbcac77	> 50	pre	Reduction Mammoplasty	0	caucasian	none
GSM7500417hbcac108	> 50	pre	Reduction Mammoplasty	0	caucasian	none
GSM7500426hbcac117	> 50	pre	Prophylactic Mastectomy	0	caucasian	unknown
GSM7500458hbcac149	> 50	pre	Reduction Mammoplasty	0	caucasian	none

HER2 breast cancer patient tissue samples (GSE176078)

(https://www.ncbi.nlm.nih.gov/geo/query/acc.cgi?acc=GSE176078)

sample ID	Age	Cancer Grade	Cancer type	HER2	Treatment
CID3586	43	3	IDC	3+	no
CID3838	49	3	IDC	3+	no
CID3921	60	3	IDC	3+	no
CID4066	41	3	IDC	3+	no
CID45171	58	3	IDC	3+	no

A. Tissue samples ID and clinical metadata of the samples selected for this stufdy from the 126 breast samples included in the breast atlas project. The columns list the age of the women, the menopause status (pre/post), the procedure from which the tissue source was collected (reduction mammoplastiesor prophylactic mastectomies), the parity status as positive (1) or negative (0), the ethnicity , whether the woman received any prior treatment before the tissue was collected.

B tissue samples ID and clinical metadata of the samples selected in this stufdy from the 26 primary tumors included in the breast cancer atlas project. The columns list the age of the women, the grade of the tumors , the cancer type, the HER2 degree, whether the woman received any prior treatment before the tissue was collected.
